# Deciphering transcriptional regulation in human embryonic stem cells specified towards a trophoblast fate

**DOI:** 10.1038/s41598-017-17614-5

**Published:** 2017-12-08

**Authors:** Ashish Jain, Toshihiko Ezashi, R. Michael Roberts, Geetu Tuteja

**Affiliations:** 1 Bioinformatics and Computational Biology, Iowa State University, Ames, IA USA; 2 Genetics, Development and Cell Biology, Iowa State University, Ames, IA USA; 3 Division of Animal Sciences, Bond Life Sciences Center, University of Missouri, Columbia, MO USA; 4 Department of Biochemistry, University of Missouri, Columbia, MO USA

## Abstract

Differentiated human embryonic stem cells (hESC) continue to provide a model for studying early trophoblast cells (TB), but many questions have been raised regarding their true identity. Therefore, we carried out a global and unbiased analysis on previously published transcriptomic profiles for hESC differentiated to TB by means of bone morphogenetic protein-4 and inhibitors of activin A and fibroblast growth factor-2 signaling (BAP treatment). Our results confirm that BAP treated hESC (ESCd) lack a mesoderm signature and are a subtype of placental cells unlike those present at term. ESCd display a high level of expression of genes implicated in migration and invasion compared to commonly used, immortalized TB cell lines and primary cells from term placenta. Co-expression network analysis also identified gene modules involved in cell migration and adhesion, processes that are likely critical during the beginning stages of placentation. Finally, protein-protein interaction analysis predicted several additional genes that may play important roles in early stages of placental development. Together, our analyses provide novel insights into the transcriptional programs that are active in ESCd.

## Introduction

The placenta is a transient organ required for fetal development and maintenance of pregnancy. In all placental mammals, it plays a major role in the transport of nutrients, gases, waste and hormones between the mother and fetus^1^. The placenta also anchors the fetus to the uterine wall and provides immune protection^1^. Trophoblast cells (TB), a cell lineage that first emerges as a simple epithelium, called trophectoderm, at the blastocyst stage of development, is involved in each of these functions. In the case of the human, implantation quickly follows blastocyst attachment to the uterine wall^2^ and appears to involve invasive syncytial TB formed ahead of a layer of progenitor TB^3,4^. By about day 12 of pregnancy, the conceptus has moved through the uterine epithelium and into the stromal region. This syncytial mass and underlying cytotrophoblast (cytoTB) completely surround the embryo proper and are believed to serve as a primitive placenta^2^.

Within days, however, columns of cytoTB have pushed through the syncytial layer to establish primary villi, which will eventually branch, acquire cores of blood vessels and connective tissue, and create the early villous placenta^5^. These villi are covered by a different kind of syncytium, which consists of a thin multinuclear cellular layer formed from fusion of underlying cytoTB^6,7^. Some of these columns of cytoTB form anchoring villi. At their tips, cells continue to divide to form an invasive extravillous TB (EVTB) population that invade further into the uterine wall. Some also enter maternal spiral arteries to alter their blood flow characteristics.

Aberrant gene expression in TB during early development is associated with abnormal placental function, which can potentially lead to pregnancy-related complications including the early onset form of preeclampsia, intrauterine growth restriction, preterm labor, and low birth weight^8–11^. Human TB from first trimester placenta are difficult to obtain and culture^12^. As a result, several other model systems have been used to study TB development, including rodent models^1^ and immortalized cell lines established from choriocarcinoma cells and first trimester EVTB^13^. Although these models are extensively used, they each have their limitations and may not be appropriate for studying early human TB function^12,14^. To address this, over the last decade many groups have tried to reprogram human pluripotent cells into TB. Xu *et al*. first reported that human embryonic stem cells (hESC) can be differentiated into TB after being treated with bone morphogenetic protein-4 (BMP4)^15^. Since then, many groups have studied this differentiation by varying the hESC culture conditions^15–18^. Although most of the studies concluded that the differentiated cells are a subtype of TB, the true identity of these cells has been debated^19–21^. To try to address this controversy, Yabe *et al*. generated RNA-Seq data from BAP treated (cells treated with BMP4, inhibitors of activin A signaling, and inhibitors of fibroblast growth factor-2 (FGF2) signaling) hESC (herein referred to as ESCd); as well as cells derived from primary cultures prepared from term placentas^17,22^. Using principal component analysis (PCA) and a panel of TB and mesoderm markers, they determined that, while ESCd do not represent TB of term placenta, they do express a full array of most known TB marker genes^22^. Because there are limitations to using specific gene markers to determine the identity of ESCd^19,21^, we decided that it would be important to carry out an unbiased analysis of the transcriptome profile of these cells.

Accordingly, we have used the RNA-Seq data generated by Yabe *et al*. to carry out a global and unbiased analysis of ESCd and compared the transcriptional profiles of these cells with publicly available RNA-Seq data from cells of mesodermal lineage, as well as TB cell lines derived from choriocarcinomas considered to represent villous cytoTB (BeWo and JEG-3) and EVTB (HTR-8/SVneo), to better understand the identity of the ESCd. Our analyses, which included functional enrichment analysis, PCA, and differential gene expression analysis, strongly indicate that ESCd are representative of invasive TB cells. We further analyzed the relationship between the genes in ESCd by carrying out co-expression network analysis, and used protein-protein interaction networks to look deeper into the gene regulatory mechanisms and predict novel genes that may be important for early placental development.

## Results

### Genes highly expressed in ESCd are enriched for placental development genes

RNA-Seq data was previously generated for three groups of ESCd (ESCd < 40 μm, ESCd 40–70 μm, and ESCd > 70 μm) based on the size of the differentiated cells^22^. Whereas the latter was comprised largely of sheets of syncytial cells, the ESCd < 40 μm and ESCd 40–70 μm fractions contained mainly mononuclear cells and a mixture of single cells and small clumps of syncytium, respectively. Similar RNA-Seq data had been generated in parallel on cytoTB cells isolated from term placentas (PHTu) and on syncytiotrophoblast (syncytioTB) generated from such cells after 48 h culture in the presence of fetal bovine serum (PHTd)^22^. These experiments revealed that both the ESCd fractions and the TB derived from term placentas were highly enriched for a large number of TB markers, yet there were major differences in their respective RNA profiles, suggesting that they possessed distinct functional attributes.

**Figure 1:**
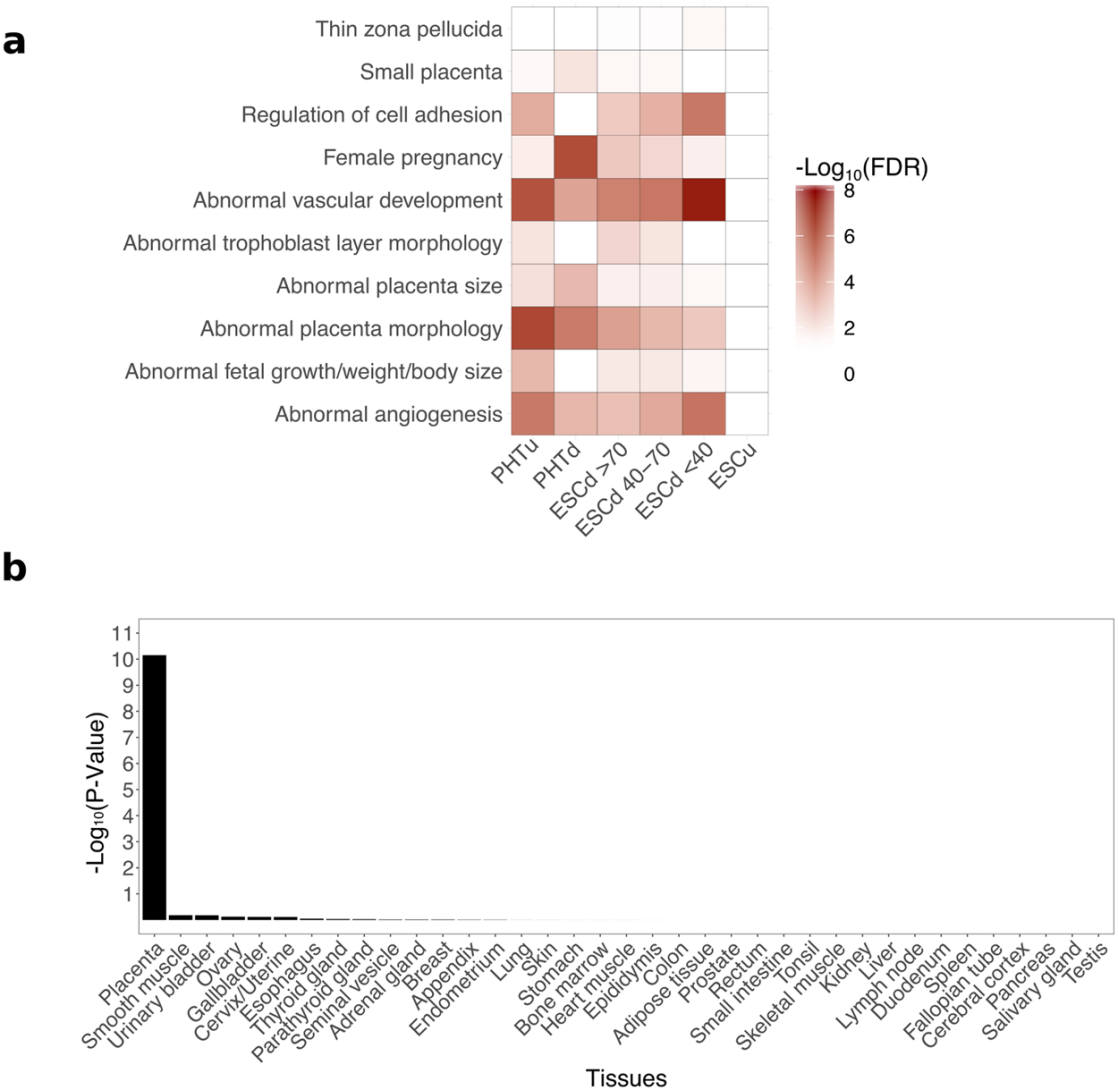
ESCd are associated with placental functions and placenta-specific genes. (**a**) Heatmap showing the enrichment (−Log_10_(FDR)) of various placental development terms. Placental terms are significantly enriched in ESCd and TB cell types. (**b**) Bar plot showing the enrichment (−Log_10_(P-Value)) of tissue-specific genes in ESCd > 70 using data from the protein atlas database. Placenta-specific genes are highly enriched in the ESCd > 70 group.

In the present study, gene expression in the above TB fractions, as well as in a number of other cell types were analyzed by procedures designed to detect functional categories of genes. The data were first normalized by using the established transcripts per million (TPM) normalization method^23^, which is designed to eliminate bias between and within samples caused by variations in transcript length, sequencing depth, and read length^24^. In order to determine if the genes most highly expressed in ESCd were associated with placental functions, the 1,000 most highly expressed genes from these cells were selected and analyzed by using the Genomic Regions Enrichment of Annotations Tool (GREAT) to determine which functional categories were enriched in each gene set^25^. The analysis revealed a significant enrichment of placental development terms, including, “abnormal placenta morphology” and “abnormal angiogenesis” in all three ESCd fractions (Fig. 1a). These terms also showed significant enrichment in top 1,000 gene lists generated from PHTu and PHTd from term placental cytoTB, but not in undifferentiated embryonic stem cells (ESCu).

We then determined if typically up-regulated placental transcripts were enriched in the list of top 1,000 genes using data from the human protein atlas database^26^, a source providing gene expression data across different tissues, including the placenta. A gene is categorized as tissue-specific if its expression level is five-fold higher in one tissue, or a small group of tissues, compared to the expression of other tissues (see Methods). For each set of tissue-specific genes, we calculated the significance of occurrence of those genes in the top 1,000 gene lists described above. We found that only placenta-specific genes were significantly enriched in each of the ESCd group gene lists (Fig. 1b and Supplementary Fig. S1a–b). As expected, the PHTu and PHTd top 1,000 gene lists also showed a significant enrichment of placenta-specific genes (Supplementary Fig. S1c–d), while the ESCu did not (Supplementary Fig. S1e). Together, these analyses provide further support that after BAP treatment, hESC differentiate into a subtype of placental cells.

### Global expression profile in ESCd are not similar to expression profile in mesoderm cells

**Figure 2:**
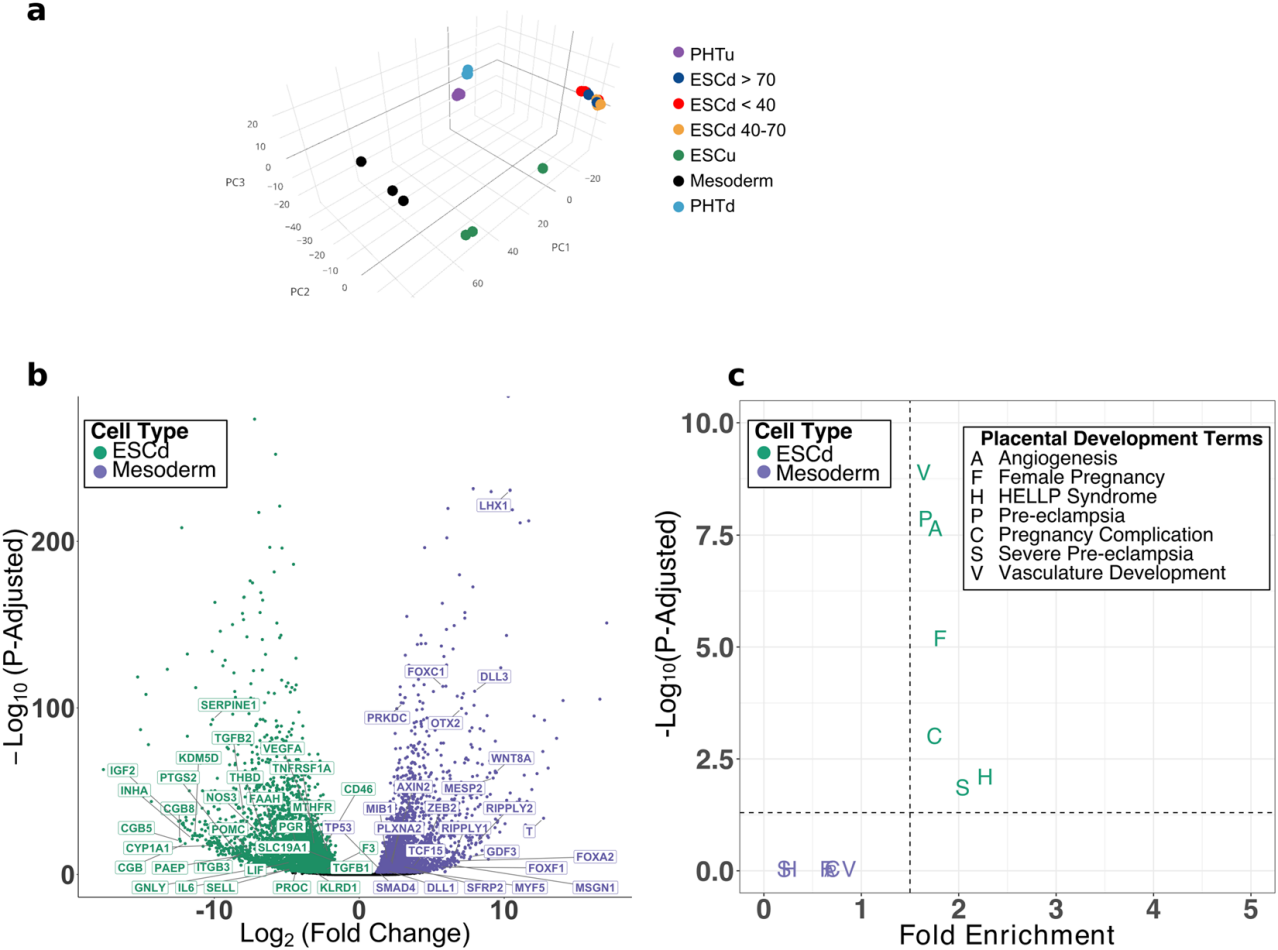
Comparison of ESCd to mesoderm cells. (**a**) PCA plot based on the 1,000 most highly expressed genes in ESCd. The ESCd samples are closer to term placental cells than to the mesoderm cells or ESCu. (**b**) A volcano plot showing the significantly upregulated genes (P-Adjusted ≤ 0.01) in ESCd (fold-change ≤ −2) and mesoderm (fold-change ≥ 2). The “pregnancy complication” and “somitogenesis” term genes upregulated in ESCd and mesoderm, respectively, are also marked in the plot. (**c**) Plot showing the enrichment of placental development terms in genes upregulated in ESCd or mesoderm. The dotted lines represent the thresholds for FDR (0.05) and fold enrichment (1.5) of the terms.

To determine if ESCd share similarities with mesoderm cells as has been suggested previously, the gene expression profiles of these cell types were compared. Mesoderm cell transcriptome profiles were previously generated by differentiating hESC towards cardiomyocytes, and harvesting cells on day two, obtaining cells representative of mesoderm^27^. We performed PCA on the 1,000 most highly expressed genes from the three ESCd groups and plotted the first three principal components, comprising of ~93% cumulative variance. The principal components show that ESCd groups cluster more closely with term placental cells (PHTu and PHTd) than with the mesoderm cells and ESCu (Fig. 2a).

Differential expression analysis between ESCd and mesoderm cells was then carried out using DESeq2^28^. For this analysis, we increased statistical power by treating the three ESCd groups (ESCd < 40 μm, ESCd 40–70 μm, and ESCd > 70 μm) as biological replicates, because the groups only had between 5 and 44 differentially expressed genes (DEGs) between them (Supplementary Fig. S2). This analysis identified a total of 7,614 DEGs between the mesoderm and ESCd groups, of which 4,441 were upregulated in ESCd and 3,173 were upregulated in mesoderm cells (Fig. 2b). When functional enrichment analysis was performed, many pregnancy-associated terms were specifically enriched in genes upregulated in ESCd, including “pre-eclampsia”, “pregnancy complication”, and “female pregnancy” (Fig. 2c). Conversely, genes upregulated in mesoderm cells were specifically enriched for mesoderm development terms, such as “abnormal mesoderm development” and “somitogenesis” (Supplementary Fig. S3). The genes from the “pregnancy complication” and “somitogenesis” terms that were upregulated in ESCd and mesoderm cells, respectively, are individually labeled in Fig. 2b. These results strongly support the argument that ESCd exhibit functional similarities to placental cells and little functional resemblance to mesoderm.

### Invasion genes are upregulated in ESCd compared to commonly used TB cell lines

**Figure 3:**
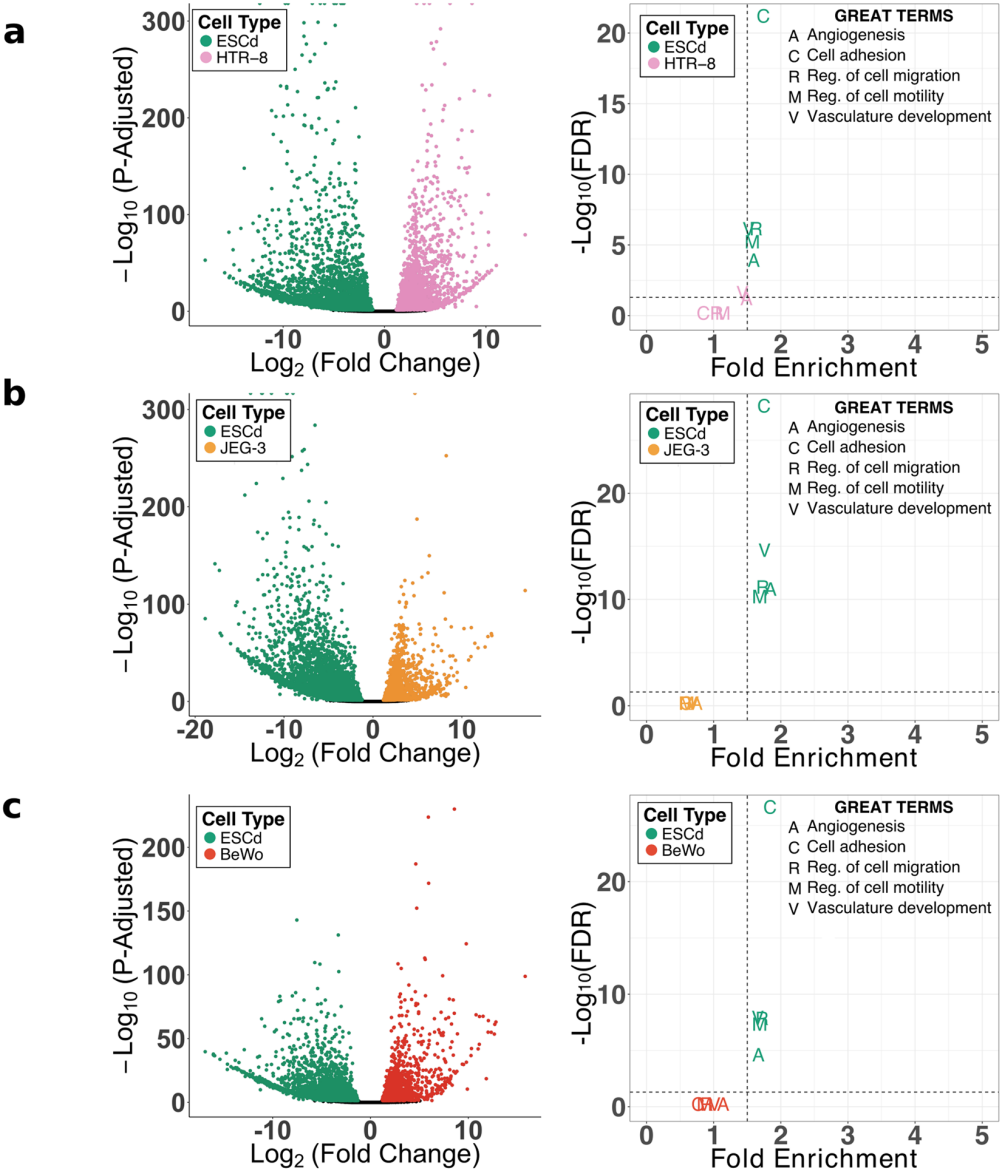
Comparison of ESCd with different placental cell lines. Volcano plots showing the differentially expressed genes between ESCd and HTR-8/SVneo cells (**a**), JEG-3 cells (**b**), and BeWo cells (**c**). The enrichment for gene ontology terms related to cell migration and invasion are also plotted for the upregulated genes in the respective cell lines in the right panels. The dotted lines represent the thresholds for FDR (0.05) and fold enrichment (1.5) of the terms.

Yabe *et al*. hypothesized that the ESCd, i.e. hESC differentiated under BAP conditions, most likely corresponded to the primitive, invasive TB that surround the embryo proper as the conceptus establishes itself in the wall of the uterus^22^. We therefore compared the gene expression profiles of ESCd to TB cell lines that have been classically used to model aspects of TB invasion^29^. Surprisingly, when compared to JEG-3^30^, BeWo^31^, and HTR-8/SVneo cells^32^, genes upregulated in ESCd were enriched for cell migration and invasion terms (Fig. 3 and Supplementary Table S1). These terms include “vasculature development”, “cell adhesion”, “angiogenesis”, and “regulation of cell motility”. While most of the genes implicated in invasion were expressed in the immortalized placental cell lines, they were expressed to a lesser extent than in the ESCd. These results are also supported by PCA (cumulative variance ~81%) in which ESCd and placental cell lines did not cluster together (Supplementary Fig. S4).

### Identification of placental gene clusters in ESCd by co-expression network analysis

The previous analyses demonstrated that genes associated with placental development and cell invasion were highly expressed in ESCd, but they did not provide information on how the various genes were linked together in molecular networks. To study this aspect of the ESCd phenotype, we performed a weighted correlation network analysis (WGCNA)^33^ to distinguish gene expression networks that were either shared between all the placental cell models, including PHTu and PHTd, or were unusually upregulated in ESCd. A total of 26 biological replicates (after processing and combining 38 RNA-Seq datasets, see Methods) from ESCd, PHTu, PHTd, BeWo, JEG-3, and HTR-8/SVneo were used to carry out co-expression network analysis. The top quartile of genes (4,815 genes) were selected based on the variance in gene expression across datasets, permitting the identification of 16 gene co-expression clusters (M1 through M16). Then, the first principal component, also known as the module eigengene, of each gene module was calculated and used to identify cell type specific modules, of which six (M5, M6, M13, M14, M15, and M16) were significantly and positively enhanced in the three ESCd fractions (Supplementary Fig. S5). Genes for each of these six modules are provided in Supplementary Data S1.

**Figure 4:**
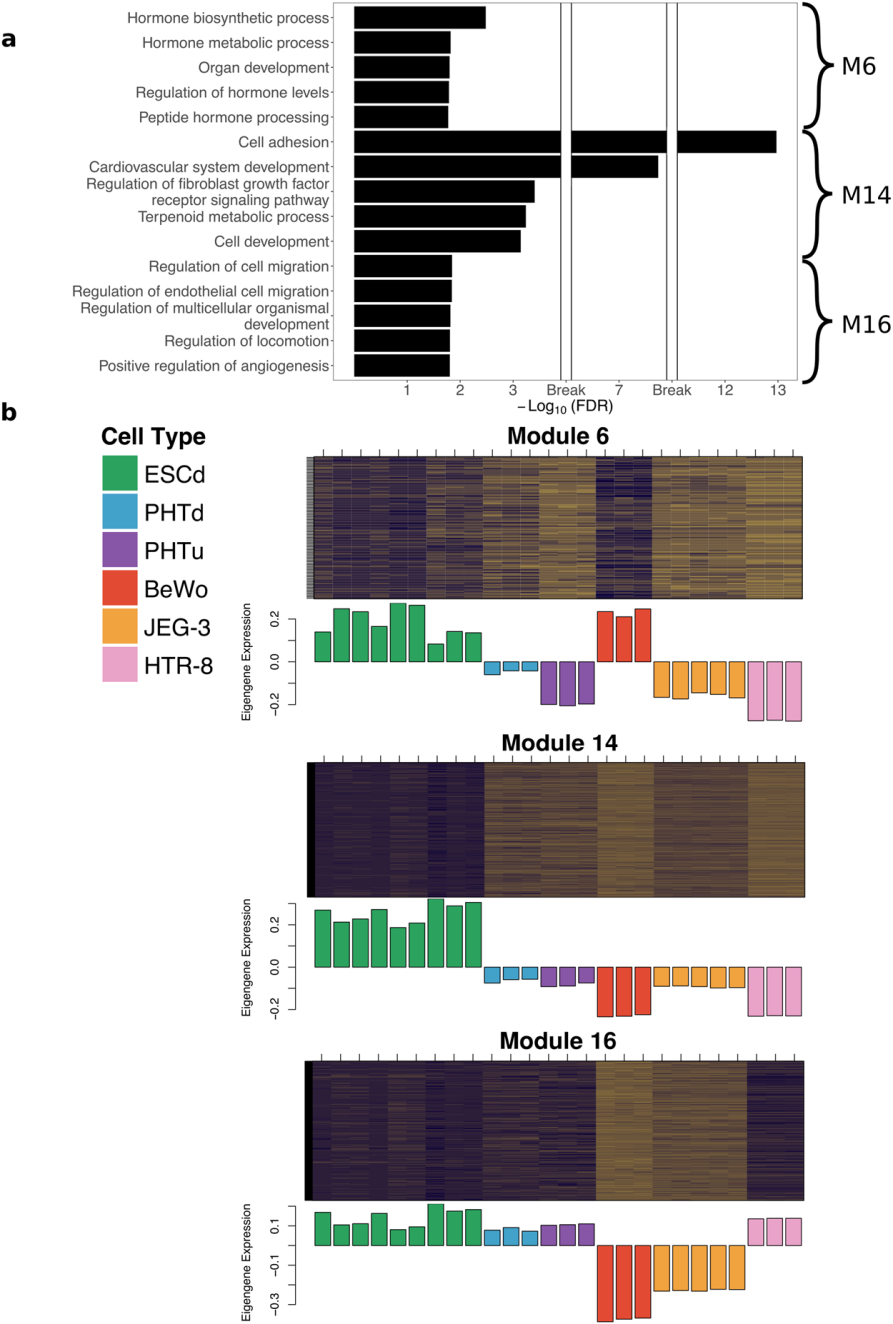
ESCd co-expression modules. (**a**) Bar plot showing the top five enriched gene ontology terms in modules M6, M14, and M16. Hormone and metabolic terms are enriched in module M6, whereas invasion and adhesion terms are enriched in modules M14 and M16. (**b**) Heatmap showing the expression of genes in co-expression modules M6, M14, and M16. The purple and brown colors represent the high and low expression of the genes in the datasets respectively. The bar plots below each heatmap show the eigengene expression in different cell types.

Interestingly, three of these modules (M6, M14, and M16), when analyzed by GREAT, showed significant enrichment of terms related to placental development (Fig. 4a and Supplementary Data S2). Modules M14 and M16 were also enriched for migration and invasion associated terms, such as “cell adhesion” (M14), and “regulation of cell migration” (M16), and for a significant number of genes implicated in the preeclampsia phenotype based on the Osborne disease ontology^34^, which is included in GREAT (False Discovery Rate (FDR) = 0.009 in M14 and FDR = 0.0005 in M16). Finally, the eigengene of module M14 had a positive and significant correlation only with the ESCd fractions, whereas M16 also had a positive correlation with HTR-8/SVneo and term placenta cells (Fig. 4b and Supplementary Fig. S5). This information suggests that genes in module M14 might be involved in a novel gene regulatory network important for early placental development.

Module M6 had enrichment for hormone and metabolic processes terms, which are some of the processes associated with syncytioTB^35^. In addition to that, the M6 module eigengene also showed a positive correlation with gene expression in BeWo cells, a cell line that has been extensively used to model events leading to the formation of syncytial TB^36^ (Fig. 4b and Supplementary Fig. S5). Together, these analyses support the hypothesis that ESCd represent precursors of, as well as differentiated syncytioTB, and demonstrate the ability to capture different TB functions in one model system^22^.

To further investigate the cell-type specificity of the genes in ESCd-associated modules, we compared their expression levels in ESCd and ESCu. We found that the expression of genes in each of modules M6, M14, and M16 is significantly higher in ESCd (P-Value < 2.2e-16) compared to ESCu (Supplementary Fig. S6). We also found a significant enrichment (P-Value < 1e-4) for genes upregulated in ESCd (ESCd vs. ESCu) in these modules by using a randomization test (Supplementary Fig. S6) (see Methods). These results provide additional evidence that genes in modules M6, M14, and M16 are important in ESCd.

### Protein-Protein Interaction (PPI) network analysis of co-expressed modules

**Figure 5:**
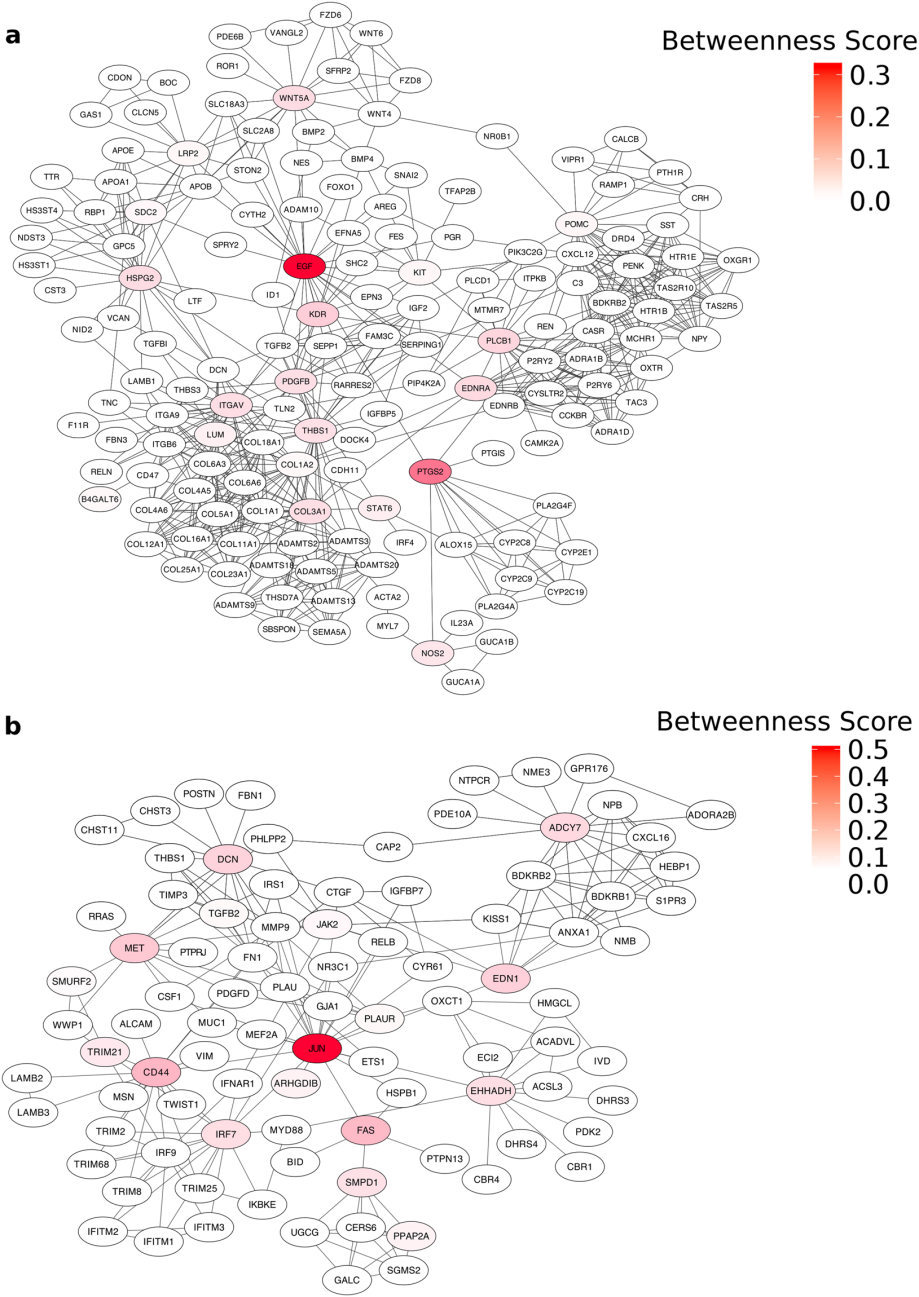
PPI Network showing bottleneck gene connections. Networks showing the predicted protein-protein interactions of the bottleneck genes and their neighbors for module M14 (**a**) and M16 (**b**). The color of the node represents the betweenness score of the gene.

In order to determine how genes within each of the placental co-expression modules may interact with one another, and to identify the key regulators within each module, the STRING database was used to construct PPI networks^37^ (see Methods). The concept of “betweenness centrality” was employed to identify those genes most likely to be essential components of the network. Betweenness centrality for a gene (node) in a biological network is defined as the number of shortest paths (between two other nodes) that pass through that node^38^. Because genes with a high betweenness control flow of information in a network, they have been called “bottleneck” genes^38^. Here, we ranked genes in the PPI networks by their betweenness score, and then defined bottleneck genes as representing the top 5% for each network (Supplementary Data S3). The bottleneck genes and the genes with which they are predicted to interact are shown in Fig. 5 and Supplementary Fig. S7. Most of the bottleneck genes identified by this analysis have been implicated in placental development, especially as it is related to TB, migration, and invasion (Table 1). Many bottleneck genes, such as *ERBB2*, *EDNRA*, *NOS2*, *POMC*, *CD44*, *FAS*, *MET*, *EDN1*, *SMPD1* have been implicated in TB invasion or in preeclampsia, but do not have a well-characterized function in early placental development. Finally, we also identified five genes (*PLCB1*, *LUM*, *ADCY7*, *IRF7*, and *EHHADH*) that we predict to be important for pregnancy disorders and placenta development, although such links remains to be established.

**Table 1:** List of bottleneck genes in the PPI networks of the ESCd co-expression modules with their function in placental development.

Bottleneck Genes	Module	Function in Placental Development	Reference
ERBB2	M6	Implicated in TB invasion and differentiation.	^59^
MMP2	M6	Involved in the invasion and proliferation of TB cells during first trimester of pregnancy.	^60,61^
EGF	M14	Increases invasive capacity of first trimester cytoTB cultures.	^62^
PTGS2	M14	Hypomethylated in term placenta.	^63^
KDR	M14	Receptor for Vascular Endothelial Growth Factor. Reduced expression in preeclamptic and preterm birth placentas.	^64^
PLCB1	M14	No known role in placenta.	NA
EDNRA	M14	Endothelin and its receptors are implicated in preeclampsia.	^65^
WNT5A	M14	Regulates the growth and development of early TB cells.	^66^
HSPG2	M14	Involved in TB cell invasion.	^67^
ITGAV	M14	Mutant mice have abnormal labyrinth layer development.	^68^
COL3A1	M14	High expression level during placental development in mouse.	^69^
PDGFB	M14	Regulates development of the labyrinthine layer in mouse placenta.	^70^
THBS1	M14	Hyperexpression in placenta is associated with disorders in placental villi maturation and branching in gestosis.	^71^
NOS2	M14	Promotes TB invasion.	^72^
STAT6	M14	Part of the signaling pathway that is involved in the proliferation of TB cells during pregnancy.	^73^
LUM	M14	Involved in cell migration, proliferation, and differentiation. No known role in placenta.	^74^
KIT	M14	Expressed in placental tissue during human pregnancy.	^75^
POMC	M14	Differentially methylated in preeclamptic placenta.	^76^
LRP2	M14	Implicated in regulation of maternal-fetal transport during pregnancy.	^77^
JUN	M16	It is a major component of activator protein 1 which helps in the invasion of the TB cells.	^78,79^
CD44	M16	Implicated in TB invasion and angiogenesis in the placenta.	^80^
FAS	M16	A polymorphism in this gene is associated with preeclampsia.	^81^
MET	M16	Implicated in TB differentiation.	^82^
EDN1	M16	Implicated in pathogenesis of hypertension in preeclampsia.	^65^
DCN	M16	Involved in TB cell migration.	^83^
ADCY7	M16	Involved in the Endothelin 1 signaling pathway. No known role in placenta.	^45^
IRF7	M16	Involved in innate immune response to viral infections. No known role in placenta.	^46^
EHHADH	M16	No known role in placenta.	NA
SMPD1	M16	Reduced activity in preeclamptic placentas.	^84^

## Discussion

In this study, we analyzed RNA-Seq data from BAP treated hESC to further understand functional properties of the cells. Previous studies largely focused on sets of gene markers to establish the identity of ESCd, which could lead to biased data interpretation. In the present study, we carried out several analyses to identify significant gene groupings, in an attempt to better understand how the ESCd might be used to model TB cells as they exist during the formation of the human placenta. Our results showed that the 1,000 most highly expressed genes in the ESCd are enriched with multiple placental development terms, and are also enriched for placenta-related disease ontology terms that included “pregnancy complication” and “pre-eclampsia”. Furthermore, we showed that previously annotated placenta-specific genes are significantly enriched among the highly expressed gene sets from ESCd and that no other tissue-specific genes indicative of the three main germ cell lineages are so enriched. Through use of PCA, we found that ESCd are somewhat related to other placental cell types but bear no resemblance to mesoderm cells derived from hESC. To confirm that, while ESCd are closer to term placental cells than mesoderm cells, they are substantially different, we also carried out PCA on ESCd, ESCu, PHTu, PHTd, and syncytioTB microdissected from term placenta^39^ (Supplementary Fig. S8). Finally, when we compared ESCd to three commonly used placental cell lines: HTR-8/SVneo, BeWo, and JEG-3, we found that genes upregulated in ESCd were enriched for terms associated with cell invasion. These results provide strong evidence that after BAP treatment hESC differentiate into a subset of invasive placental cells, and may provide good model for studying TB functions, including invasion, as has been demonstrated in a number of other studies^40–44^.

Co-expression network analysis has provided an insight into gene regulatory mechanisms operating in ESCd by identifying groups of genes that are likely to work together to regulate specific functional processes. Genes that made up two (M14 and M16) of the six modules we identified were enriched with different GO Biological Process terms related to cell migration and invasion. It should also be noted that the M14 module eigengene is positively correlated only with ESCd, indicating that the genes in this module may be a part of a unique gene regulatory network that cannot be captured by studying either term placental cells, such as the primary PHTu and PHTd, or the BeWo, JEG-3, and HTR-8/SVneo cell lines. We also found that the eigengene of the M6 module has a positive correlation with BeWo cells, which have been used to model syncytial fusion^36^. This module is also enriched with “peptide hormone processing” and “hormone metabolic process” terms, which are some of the processes associated with syncytioTB. Together, the cell adhesion, cell migration, and hormone process gene co-expression networks identified support previous studies that speculated that ESCd may represent primitive, invasive EVTB of the early stage conceptus^22^.

We further explored these co-expressed modules using PPI data from the STRING database. This analysis showed very dense PPI networks for modules M14 and M16, which contain genes functioning in cell adhesion and cell invasion, processes that have been extensively studied in cancer cells. The PPI network for module M6 was much less dense, possibly because there are many understudied genes in this module. Based on betweenness centrality, we found that many bottleneck genes in the PPI network either play an important role in the development and maintenance of early TB cells or are implicated in preeclampsia (Table 1). Moreover, we discovered five novel genes which have not been directly implicated in placental development or pregnancy disorders, but are known to be involved in relevant pathways including the endothelin 1 signaling pathway (adenylate cyclase 7; *ADCY7*)^45^ and immune response (interferon regulatory factor 7; *IRF7*)^46^. Interestingly, we found that four out of the five novel genes have high expression (Fragments per kilobase of transcript per million mapped reads (FPKM) > 5) in E7.5 or E9.5 mouse placental tissue^47^ (Supplementary Fig. S9), providing further support for the possibility that these novel genes identified by co-expression analysis should be further investigated for their putative role in placental development and pregnancy disorders.

The above discussion raises the question as to the precise nature of ESCd and their relationship to trophectoderm, the source material for all placental trophoblast. Our assumption is that ESCd represent the syncytial and cytoTB layers that surround the embryo proper soon after implantation begins, i.e. from about days 8–12, during the second week of pregnancy before the placental villi have emerged, a stage that is no longer available for experimentation^2^. If this supposition is correct, we would predict that the differentiation pathway passes through a trophectoderm-like state early after the onset of differentiation^18^, while the ESCd would possess a transcriptome somewhat intermediate to the trophectoderm of the blastocyst before it implants at around day 7 and the villous trophoblast that begins to emerge towards the end of the second week post-coitus. Although transcriptome data are not available for the latter, they are available from single cell RNA-Seq analyses performed on human blastocysts on days 5, 6, and 7 of preimplantation development, i.e. from the time the blastocyst first forms until the time it would normally initiate implantation^48^. At day 5, PCA of the sequencing data enabled cells to be assigned to either the inner cell mass (epiblast plus emerging extraembryonic endoderm) or the trophectoderm. Over 2,000 genes were differentially expressed between the two classes of cells, and, by day 7, this value increased to over 3,000. By using the differential expression analysis of trophectoderm against other lineages, a list of 100 trophectoderm-specific genes was generated^48^. Of these trophectoderm-specific genes, most are expressed in ESCd (Supplementary Fig. S10 and Supplementary Data S4). A few trophectoderm-associated genes (e.g. *CYP26A1*, *SLC34A2*, and *MYLPF*) had low expression in ESCd relative to trophectoderm. Genes encoding two transcription factors associated with trophoblast specification, *GATA2, GATA3*, were among those close to the top of the trophectoderm ranking and were also expressed robustly in ESCd. There were also two distinguishable populations of trophectoderm cells in day 6 and day 7 human blastocysts, one representing mural trophectoderm, and the other polar trophectoderm^48^. The latter, neighboring the inner cell mass, represents the region that attaches to the uterine epithelium and the source of the invasive cells, believed to be syncytial, that may allow the conceptus to implant in primates^49–51^. Again, genes with a highly significant bias in expression in polar trophectoderm (top 100 at day 7) are also expressed in ESCd (Supplementary Fig. S10 and Supplementary Data S4). These data suggest that both the polar trophectoderm and the ESCd are active in placental hormone production (*CGA* and placental growth factor, *PGF*) and formation of syncytioTB (*GCM1*, *OVOL1*, *ERVV-1*, and *ERVV-2*). Together, these two sets of data do not prove, but are consistent with a close ontological relationship between implanting trophectoderm and ESCd.

In summary, our analyses provide evidence in support of the hypothesis that BAP treated hESC represent early invasive syncytial TB. The gene co-expression analysis highlighted networks in ESCd that may provide insight into protein-protein interactions relevant for early placental development. The genes identified from this analysis should be further studied to understand their role in placental development.

## Methods

### RNA-Seq data processing

We used publicly available RNA-Seq datasets downloaded from the Gene Expression Omnibus (see Supplementary Table S2). First, the quality and the adapter content of each dataset was evaluated using FastQC^52^. The low-quality reads and the adapter content identified from FastQC were filtered using Trimmomatic^53^. The filtered reads were aligned to the reference human genome (hg19) using HISAT2^54^, and were further filtered to remove reads that map to the mitochondrial genome. The number of reads that aligned to each protein coding gene were counted using the htseq-count tool from the HTseq software package^55^. RNA-Seq data generated from PHTu and PHTd using the same culture conditions on the same day were treated as technical replicates, and combined by adding raw read counts. For JEG-3 RNA-Seq data, each data set from wild-type samples were considered a biological replicate. For other samples, we combined the technical replicates by adding raw read counts. We normalized gene counts for each biological replicate by converting them into log transformed TPM values. TPM values were calculated by normalizing the read count first by the gene length and then by the sequencing depth^23^.

### Human Protein Atlas analysis

To evaluate the number of placenta-specific genes that are highly expressed in our samples, we used the human protein atlas database (version 16.1). This database contains mRNA expression data across different tissues including the placenta^26^. The genes in the database are categorized based on tissue-specific expression, and we used their representational state transfer application programming interface to extract this information. The database contains the expression details of 19,628 genes of which 7,835 are categorized to have some level of tissue specificity (protein atlas categories: tissue enriched, group enriched, and tissue enhanced groups). Out of those 7,835 tissue-specific genes, 354 have tissue specificity in the placenta. We took the total number of genes as the population size, number of placenta-specific genes as number of successes in the population, number of input genes as the number of draws, and the number of placenta-specific genes found in the input genes as number of observed successes to estimate the significance of each set of tissue-specific genes using hypergeometric test.

### Differential expression analysis

DESeq2 was used for all of the differential expression analysis^28^. Genes were considered upregulated if they had an absolute fold-change ≥2 and adjusted p-value ≤ 0.01.

### Functional enrichment analysis

Genomic Regions Enrichment of Annotations Tool (GREAT)^25^ was used for functional enrichment analysis of gene sets using the following ontologies: GO Biological Process, Mouse Phenotype, and Disease Ontology. Because we were investigating gene sets, significance was assessed using the hypergeometric statistic^25^. Terms were considered significant if they had an FDR ≤ 0.05, a fold-change ≥ 1.5, and at least 5 genes from the input set.

### Weighted co-expression network construction

To construct the weighted co-expression network we used the WGCNA package in R^33^. We used gene expression data for biological replicates from ESCd, PHTu, PHTd, and the placental cell lines to construct the network. The mapping information of the cell type and dataset were stored in a binary mapping file in which the rows consist of the data points and the columns represent the cell types. After that, we retained the genes with a TPM > 1 (19,261 genes) and selected the top quartile genes (4,815 genes) based on the variance for network construction. The selected genes were used to calculate the adjacency matrix by using the signed hybrid Pearson correlation method with a soft thresholding power. The power was selected to achieve a scale free topology for the co-expression network by plotting the scale free fit and mean connectivity at different soft thresholding powers. We used a power of 16 resulting in a scale-free network (R^2^ = 0.9)^56^ that retains a good number of connections (Supplementary Fig. S11). The interconnectedness (topological overlap), which is used to cluster the genes by hierarchical clustering was calculated. We subsequently applied the dynamic tree cut method to obtain the clusters and merged the closely related clusters based on the correlation between them (correlation > 0.75) to get the final modules^57^. The modules were decomposed such that each module was represented by its weighted expression (module eigengene) in the form of its first principal component. The relationship between the modules was identified by calculating the correlation between them (Supplementary Fig. S12). We also calculated the module membership (K_ME_) for each gene, which is the correlation between the gene expression and the module eigengene. Based on the K_ME_, we assigned genes to modules when the correlation was greater than 0.75, allowing a gene to be a part of multiple modules or multiple regulatory pathways (Supplementary Fig. S13). The significance of the module in the cell type was then estimated by calculating the correlation between the module eigengene and the gene expression in the cell data types which was mapped in the traits file. Using this method, the gene modules representing the different cell types were identified (Supplementary Fig. S5).

### Randomization Tests

Randomization tests were used to determine if modules associated with ESCd had a significant number of genes upregulated in ESCd compared to ESCu. For each ESCd module, we generated 10,000 random gene modules that matched in size. For each random gene module, we counted the number of genes that were upregulated in ESCd vs ESCu. The p-value was calculated as the number of random modules that have more genes upregulated in ESCd than in the actual module (divided by 10,000).

### PPI network analysis

We used the STRING database (version 10.5) to construct PPI networks from the co-expressed genes^37^. A threshold weight of 0.7 was used for selecting the connection between two proteins. We extracted the largest connected sub-network from the PPI network and computed the betweenness using Cytoscape^58^. We defined the top 5% genes in each network as bottleneck genes based on their betweenness score (score ≥ 95 percentile).

### Abbreviations

A list of abbreviations used in this paper is compiled in Supplementary Table S3.

### Data Availability

RNA-Seq data used in this study were downloaded from the Gene Expression Omnibus (accession numbers are in Supplementary Table S2). Additional data from analyses have been made available as supplementary data files.

## Electronic supplementary material

Supplementary Material

Supplementary Data S1

Supplementary Data S2

Supplementary Data S3

Supplementary Data S4
