# Supplementary material for: Deciphering transcriptional regulation in human embryonic stem cells specified towards a trophoblast fate
Source: Sci Rep. 2017 Dec 8;7:17257. doi: 10.1038/s41598-017-17614-5 (PMC5722916; doi:10.1038/s41598-017-17614-5)
Supplement: Supplementary file 1 — Supplementary Material [file 41598_2017_17614_MOESM1_ESM.pdf]

Deciphering transcriptional regulation in human embryonic stem cells specified towards a trophoblast fate.

Ashish Jain[1,2], Toshihiko Ezashi[3], R. Michael Roberts[3,4], and Geetu Tuteja[1,2,]*

[1]Bioinformatics and Computational Biology, Iowa State University, Ames, IA, USA.
[2]Genetics, Development and Cell Biology, Iowa State University, Ames, IA, USA.
[3]Division of Animal Sciences, Bond Life Sciences Center, University of Missouri, Columbia, MO, USA
[4]Department of Biochemistry, University of Missouri, Columbia, MO

*Corresponding Author

Geetu Tuteja: geetu@iastate.edu

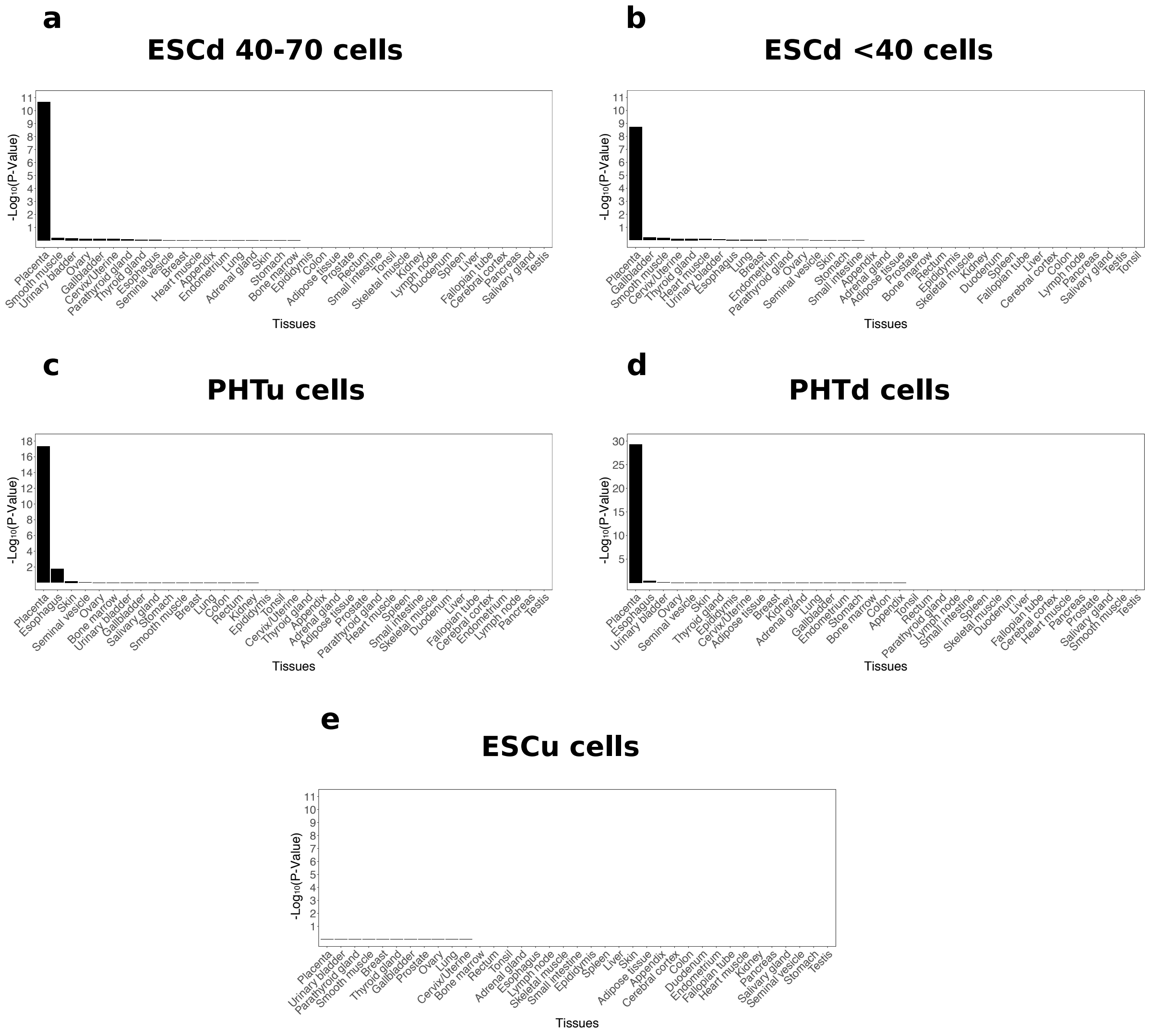

Figure S1: (a-e) Bar plots showing the enrichment (-Log$_{10}$(P-Value)) of tissue-specific genes in ESCd 40-70, ESCd <40, PHTu, PHTd, and ESCu cells using the protein atlas database. Placenta-specific genes are enriched in all cell types except ESCu.

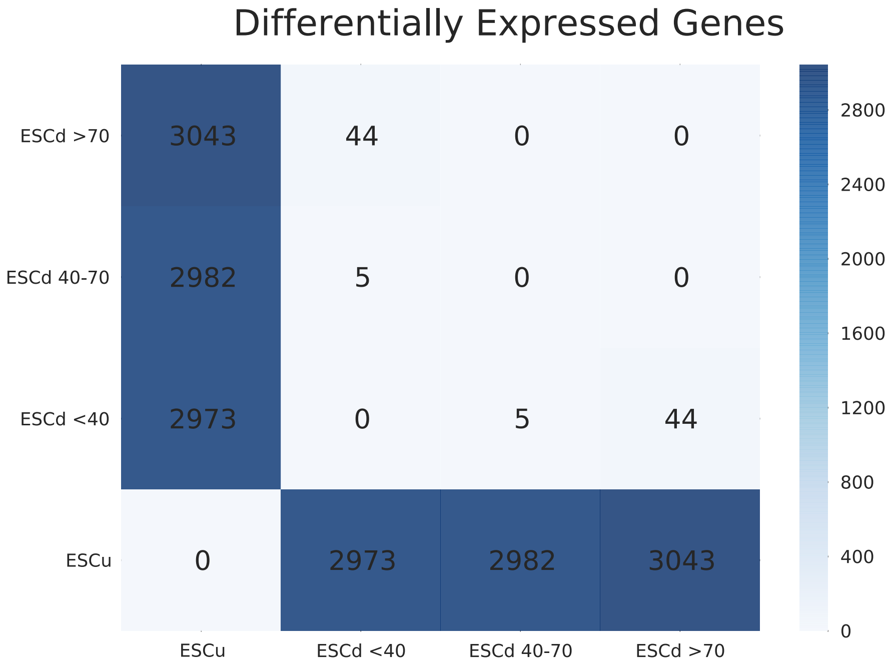

Figure S2: Heatmap showing the significant differentially expressed genes (P-Adjusted ≤0.01 and abs(Fold-Change) ≥2) between the ESCu and ESCd groups. ESCd groups have few differentially expressed genes between one another.

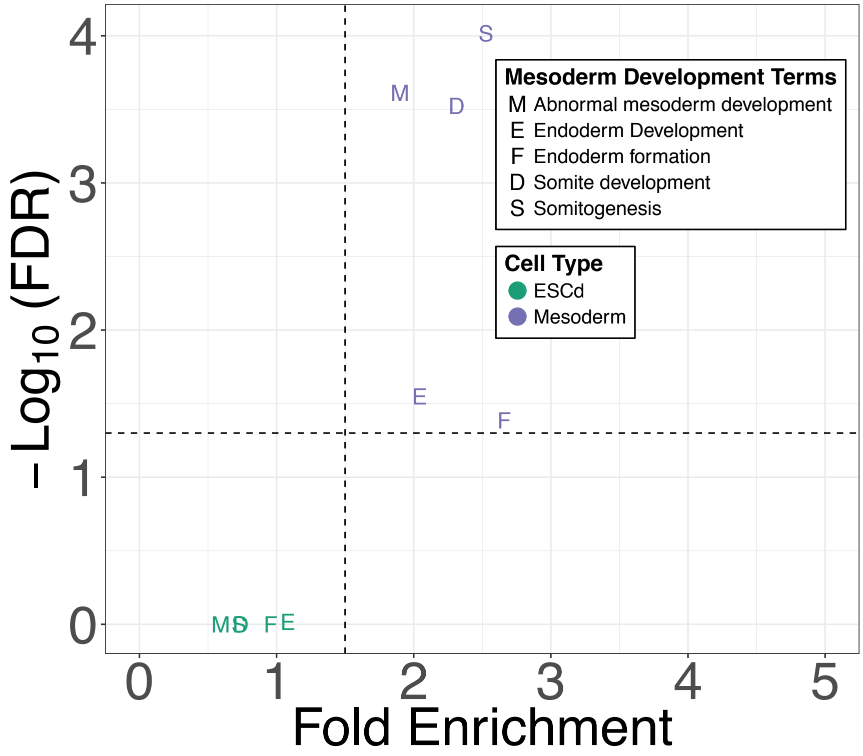

Figure S3: Plot showing the enrichment of mesoderm development terms in genes upregulated in ESCd or mesoderm. The dotted lines represent the thresholds for FDR (0.05) and fold enrichment (1.5) of the terms.

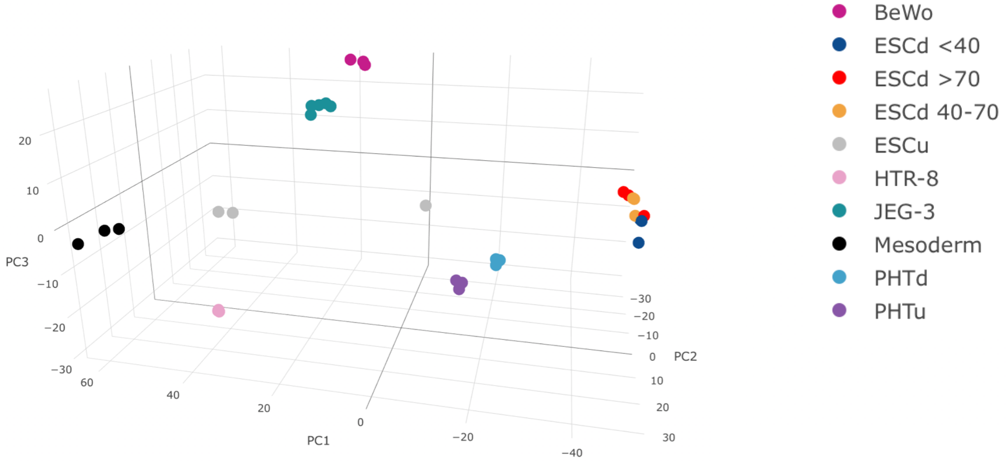

Figure S4: PCA plot based on the 1,000 most highly expressed genes in ESCd. PCA was carried out using ESCd, ESCu, placental cell lines, term placental cells, and mesoderm cells. The first three principal components are plotted (cumulative variance of ~81%).

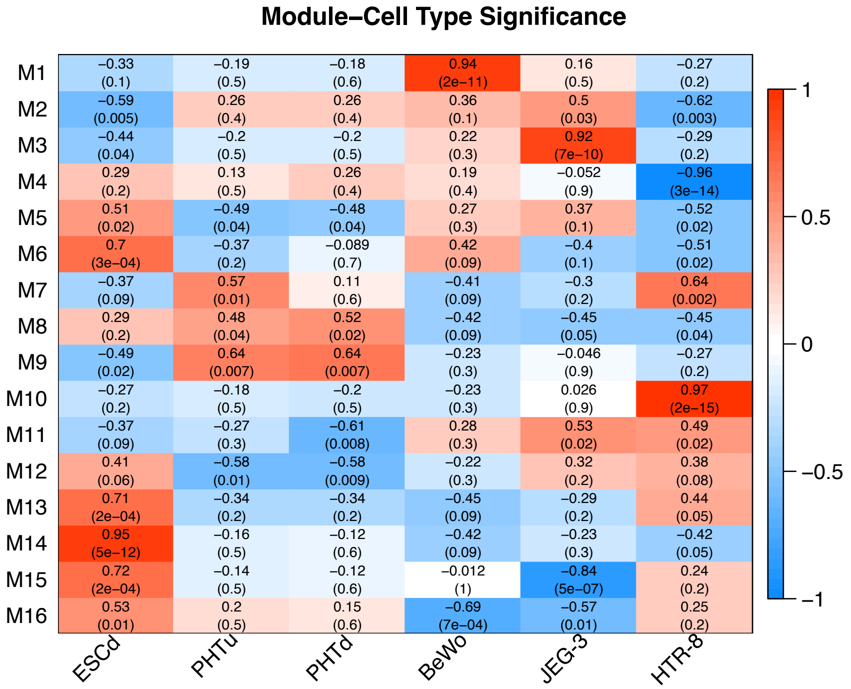

Figure S5: Heatmap showing the relationship between the co-expressed modules and cell type. Each block in the heatmap has the correlation score (top) and adjusted p-value (bottom) between the respective co-expressed module and cell type.

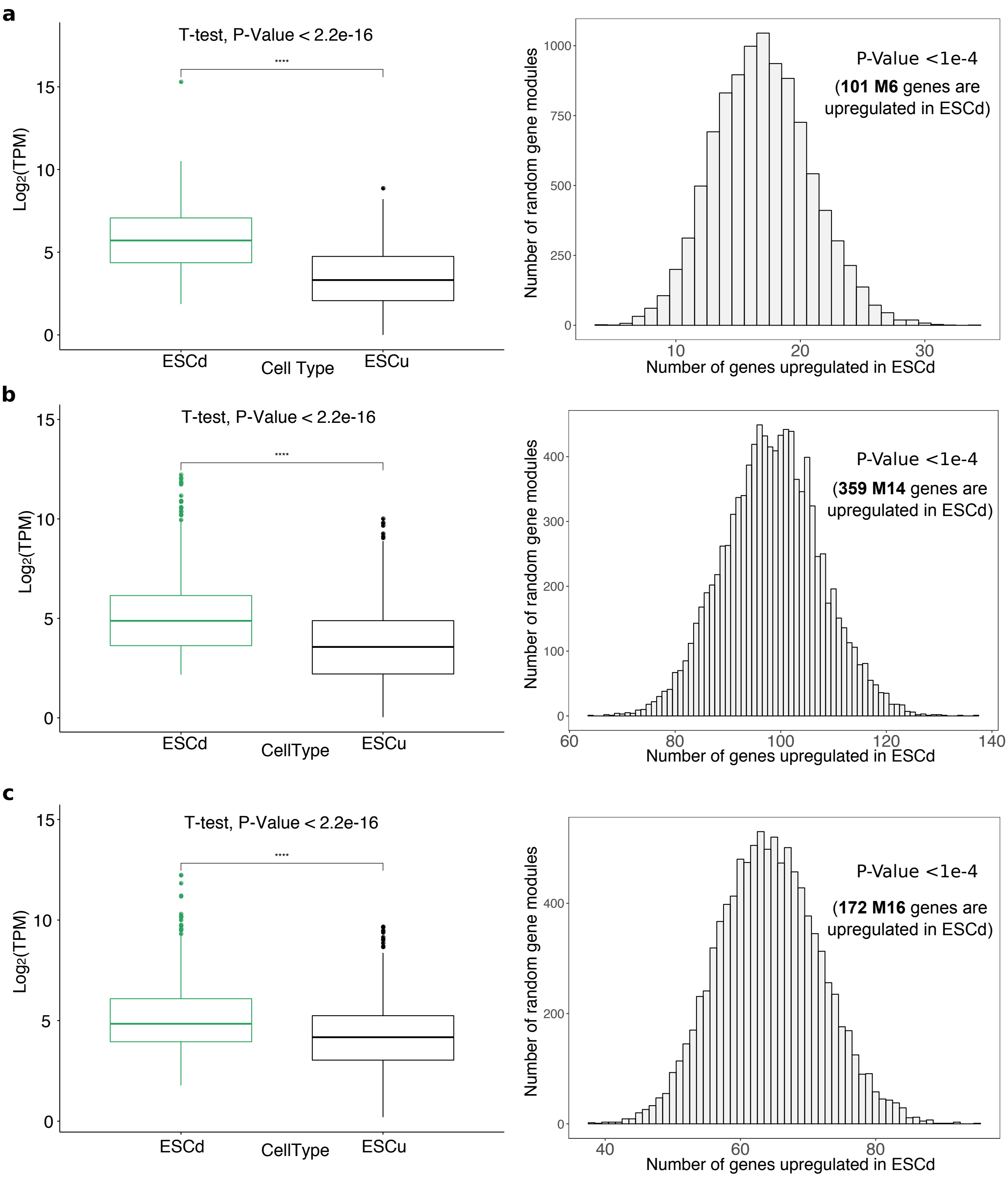

Figure S6: Box plots (left panels) showing expression values in ESCd and ESCu for genes in modules M6 (a), M14 (b), and M16 (c). Gene expression is significantly higher in ESCd. The corresponding right panels show the distribution of the number of genes upregulated in ESCd in 10,000 sets of random gene modules.

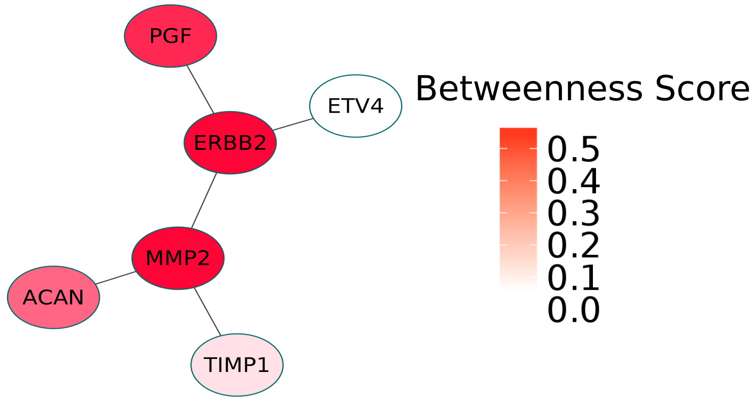

Figure S7: Network showing the protein-protein interaction between the bottleneck genes and their neighbors for module M6. The node color represents the betweenness score of the genes.

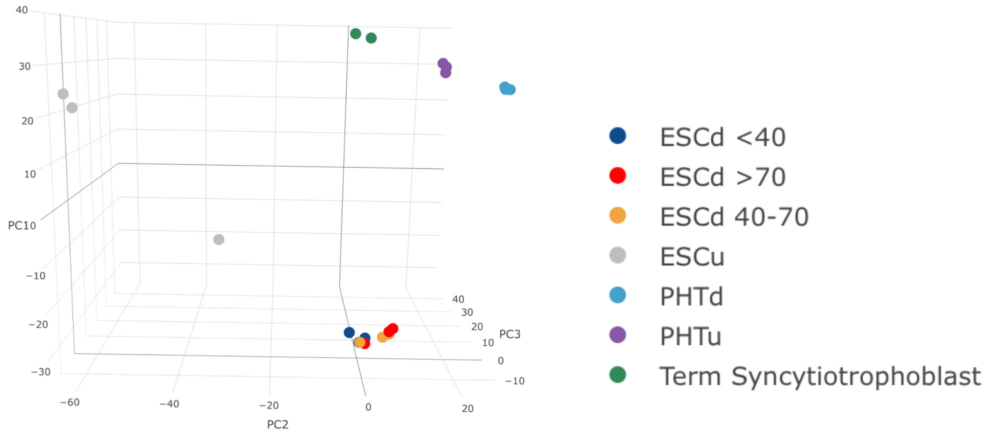

Figure S8: PCA plot based on the 1,000 most highly expressed genes in ESCd. The first three principal components are plotted with a cumulative variance of ~90%. PCA was carried out using the ESCd, ESCu, and term placenta cells.

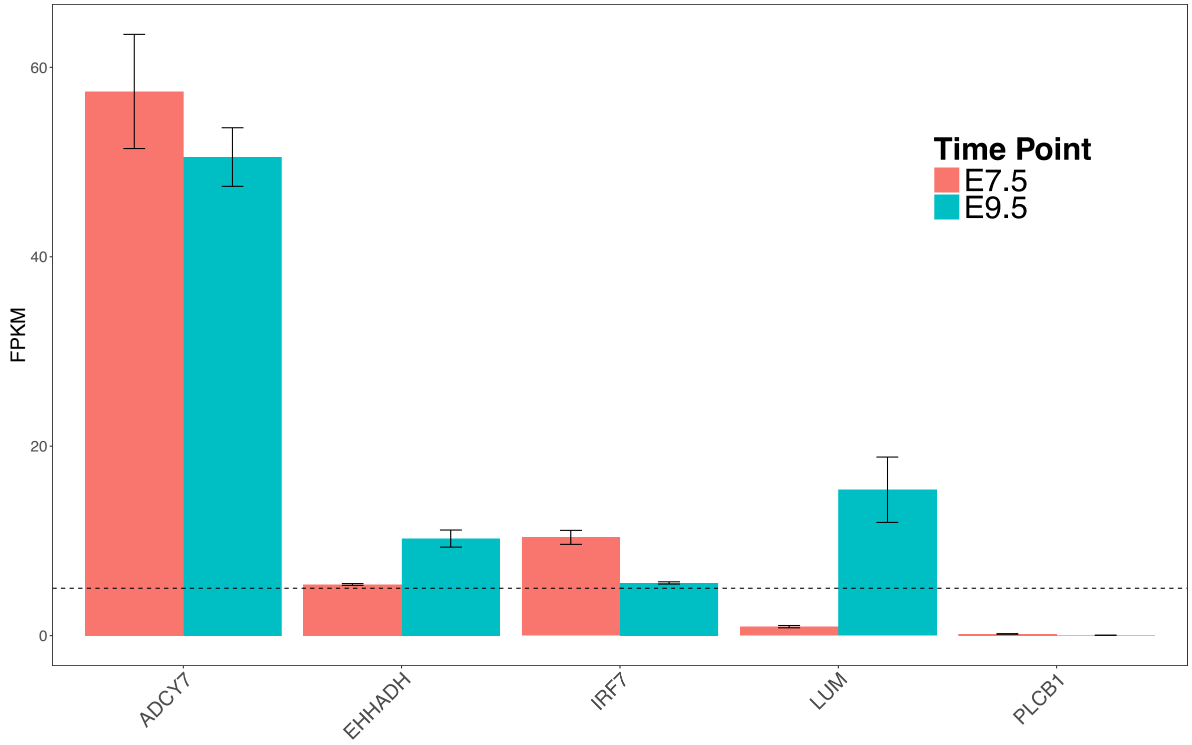

Figure S9: Plot showing the expression of the novel genes in E7.5 and E9.5 mouse placenta. The error bars represent the variance in the samples. The dotted line represents the expression level of 5 FPKM.

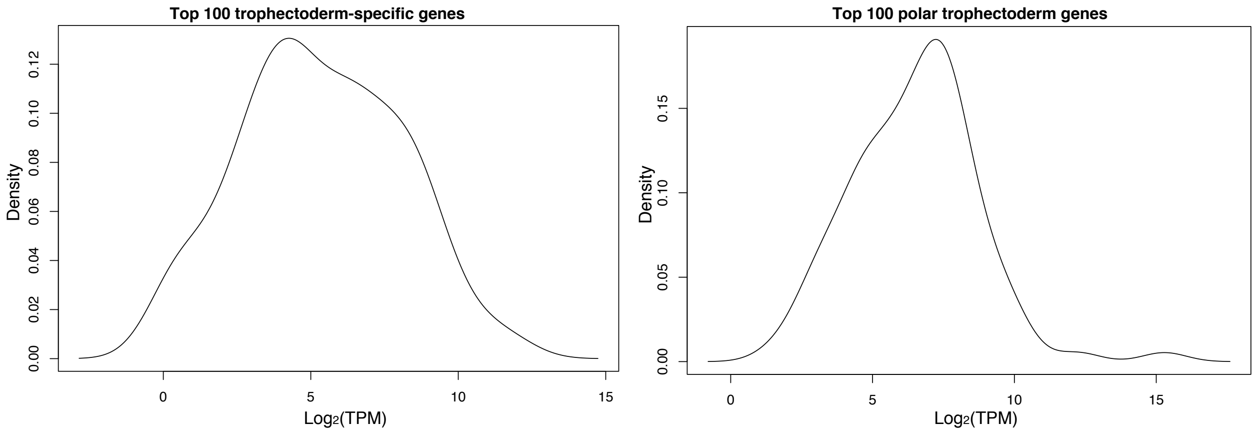

Figure S10: Distribution of Log_2(TPM) for genes that are trophectoderm specific (left) and genes that have a highly significant bias in expression in polar trophectoderm (right).

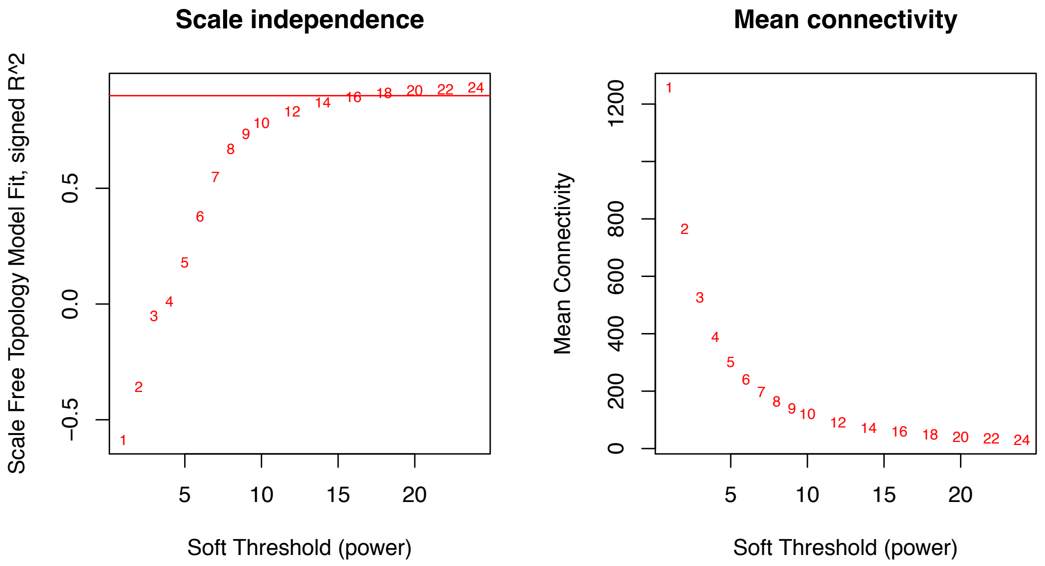

Figure S11: Plots showing the R^2 values (left panel) and mean connectivity (right panel) of various soft threshold values required for making the co-expression network scale free. The threshold for the R^2 value is 0.9, shown as the red horizontal line.

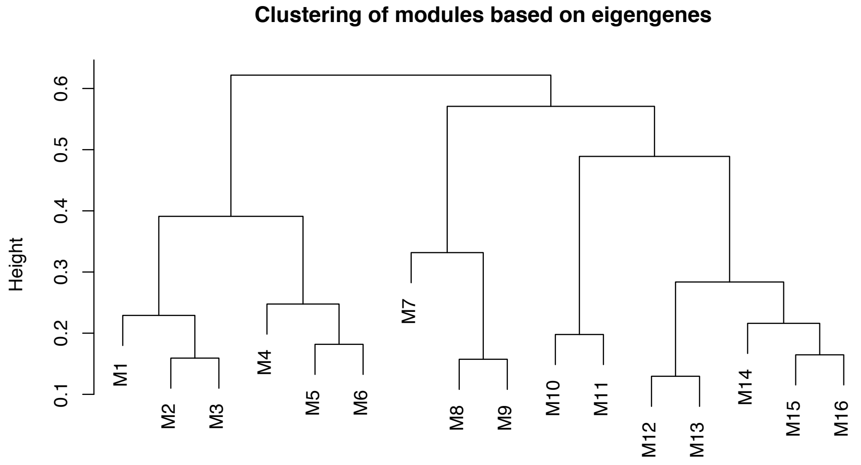

Figure S12: Dendrogram showing clustering of the modules based on the correlation between the module's eigengene expression.

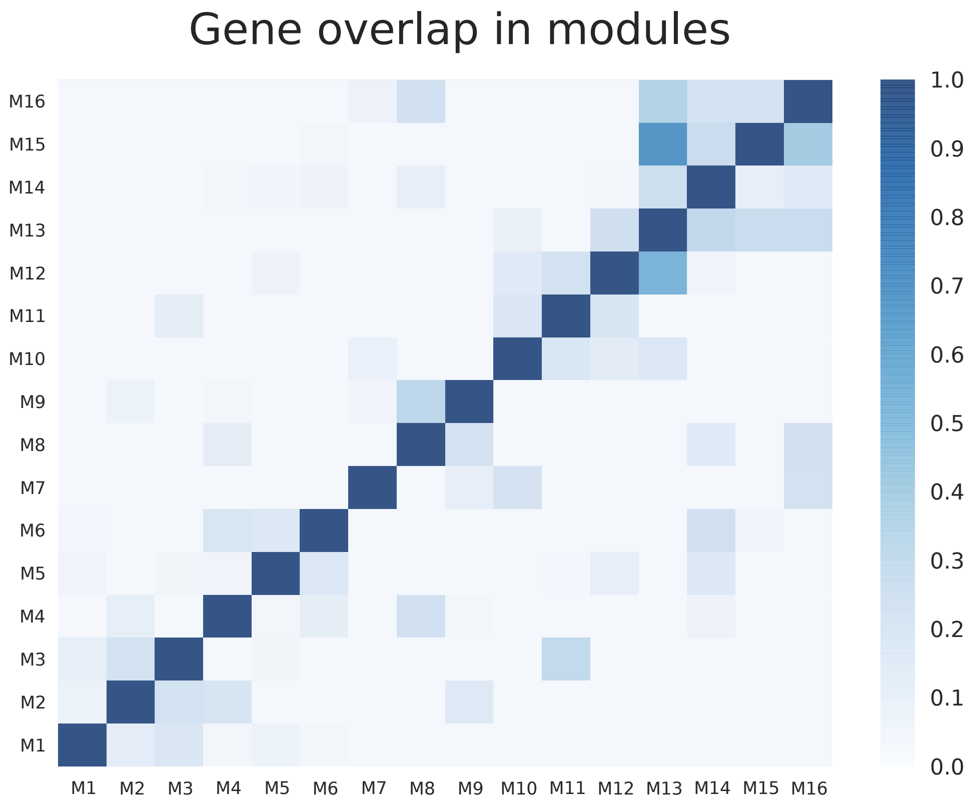

Figure S13: Heatmap showing the proportion of genes overlapping between co-expressed modules based on the module membership (K_{ME}) of genes.

Table S1:
Summary of the differential expression analysis of the placental cell lines with ESCd.

Cell Type	DEGs	Genes upregulated in ESCd	Genes downregulated in ESCd
BeWo	5478	3866	1612
HTR-8/SVneo	6516	4081	2435
JEG-3	6793	4756	2037

Table S2:
Detailed information about the RNA-Seq datasets used for analysis.

Cell Type	Study Accession	Read Length	HISAT2 Alignment (Average alignment score across datasets)
ESCd >70µm	GSE72712	50bp	82.31%
ESCd 40-70µm	GSE72712	50bp	82.09%
ESCd <40µm	GSE72712	50bp	82.28%
ESCu	GSE72712	50bp	82.56%
PHTu	GSE73016	50bp	82.10%
PHTd	GSE73016	50bp	81.49%
Mesoderm Cells (Day 2 of differentiation)	GSE64417	100bp	93.90%
BeWo Cells	GSE66962	100bp	93.90%
JEG-3 Cells	GSE79779	100bp	96.30%
HTR-8/SVneo Cells	GSE85995	75bp	96.40%
Term Syncytiotrophoblast	GSE87725	75bp	93.5%

Table S3:
List of abbreviations used in the paper.

Abbreviation	Definition
BAP	Bone morphogenetic protein-4, activin A signaling inhibitor, and fibroblast growth factor-2 signaling inhibitor treatment
BMP4	Bone morphogenetic protein-4
CytoTB	Cytotrophoblast
DEGs	Differentially expressed genes
ESCd	Human embryonic stem cells differentiated using BAP treatment
ESCd <40	ESCd that are less than 40μm in size
ESCd >70	ESCd that are greater than 70μm in size
ESCd 40-70	ESCd that are in between 40μm and 70μm in size
ESCu	Undifferentiated embryonic stem cells
EVTB	Extravillous trophoblast
FDR	False discovery rate
FGF2	Fibroblast growth factor-2
FPKM	Fragments per kilobase of transcript per million mapped reads
GREAT	Genomic regions enrichment of annotations tool
hESC	Human embryonic stem cells
K_{ME}	Module membership: correlation between the gene expression and the module eigengene
PCA	Principal component analysis
PHTd	Syncytiotrophoblast cells generated by in vitro culture of cytotrophoblast cells from term placenta
PHTu	Cytotrophoblast cells isolated from term placenta
PPI	Protein-protein interaction
SyncytioTB	Syncytiotrophoblast
TB	Trophoblast cells
TPM	Transcripts per million
WGCNA	Weighted correlation network analysis

Supplementary Data Files
Supplementary Data S1: Genes in the ESCd co-expression modules.
Supplementary Data S2: GREAT terms enriched in ESCd modules.
Supplementary Data S3: Betweenness score of the genes in the PPI networks.
Supplementary Data S4: Log_2(TPM) for TE-specific genes and top 100 polar TE genes.
